# Microbial Community Dynamics and Biogeochemical Cycling in Microplastic-Contaminated Sediment

**DOI:** 10.3390/nano15120902

**Published:** 2025-06-11

**Authors:** Xuanxuan Zhang, Dina Ding, Yinglin Liu, Zhiming Yao, Pingping Duan, Hanyu Yuan, Hanzhong Fan, Yanhui Dai

**Affiliations:** 1 Institute of Coastal Environmental Pollution Control, Key Laboratory of Marine Environment and Ecology (Ministry of Education), Ocean University of China, Qingdao 266100, China; 2 School of Environmental Science and Engineering, Suzhou University of Science and Technology, Suzhou 215009, China

**Keywords:** microplastics, sediments, microbial community, biogeochemical cycling

## Abstract

Sediments are important repositories for microplastics (MPs) which exhibit higher microbial community richness and greater diversity than corresponding aqueous phases. Recently, the effects of MPs on microorganisms in sediments have received widespread attention. This review summarizes current knowledge on how MPs alter microbial diversity, composition, function, and biogeochemical cycling in sedimentary environments. The impacts of MPs on microorganisms in sediments can be affected by several factors, including MP type, the sedimentary environment, exposure time, and exposure concentration. Generally, biodegradable MPs cause more significant changes to the microbial community structure in sediments due to degradability and high bioavailability. Short-term exposure to MPs may enhance microbial diversity, and long-term exposure may lead to a reduction in diversity. High concentrations cause more serious impacts on microbial diversity than low concentrations. MPs mainly interfere with cycles of carbon, nitrogen, phosphorus, and sulfur in the sedimentary environment by changing microbial community structure, enzyme activity, and gene abundance. In conclusion, key research gaps are pinpointed, and future research directions presented. This review provides valuable insights into the health risks and ecological responses of MPs in sedimentary environments.

## 1. Introduction

Plastics are widely used in daily life and in industry for their stability, lightness, and low cost [1]. Plastics can be further decomposed into microplastics (MPs) by physical, chemical and biological processes [2,3]. Forecasting models suggest that the amounts of MPs released into the environment could rise by 1.5–2.5 times by 2040, leading to health risks to biota and humans [4]. MPs are widespread in water environments and have been frequently detected [5]. Sediments are special regions of aquatic ecosystems that may accumulate high concentrations of MPs. Eriksen et al. [6] found that actual amounts of MPs in seawater were substantially lower than predicted amounts, with actual amounts being just 1% of the predicted values. Furthermore, Boucher et al. [7] showed that MPs in sediment accounted for 3.3% of sediment weight on plastic-contaminated beaches. All of the above results indicate that large amounts of MPs in the aqueous phase may settle into the sedimentary environment.

Microorganisms, as key biological components of sediments, participate in core ecological functions such as the carbon–nitrogen cycle [8,9], organic matter degradation [10], and pollutant transformation [11]. Their sensitivity to MP stress is directly related to the stability of the ecosystem. MPs not only provide a carbon source for the growth and metabolism of microorganisms, but also provide a substrate for colonization by microorganisms [12]. In addition, MPs can affect the diversity and composition of microbial communities. Song et al. [13] analyzed microbial α diversity in marine sediments. Their findings indicated that MPs might reduce the diversity and abundance of microorganisms in marine sediments. MPs can also affect the functions of microorganisms by altering the structure of the microbial community, the activity of related enzymes, and the abundance of genes, thereby influencing the cycling of carbon, nitrogen, phosphorus, and sulfur in the sedimentary environment [14,15,16,17]. Seeley et al. [18] found that abundance of the *amoA* gene (an enzyme gene related to ammonia oxidation) was lowest in a polyvinyl chloride (PVC) treatment group, suggesting that PVC treatment inhibited nitrification. Wang et al. [19] revealed that polylatic acid (PLA) MPs increased abundances of sulfate-reducing bacteria and sulfate-reducing genes. However, current research is still insufficient with regard to the influence mechanism of MPs on the biogeochemical cycle of sedimentary environments. Furthermore, the influencing factors of MPs on microbial communities and biogeochemical cycles in sedimentary environments remain unclear. There is still a lack of a comprehensive summary to guide further research.

This review primarily summarizes the impacts and underlying mechanisms of MPs on the colonization, diversity, and composition of microbial communities, as well as microbial ecological functions and biogeochemical cycles in sedimentary environments. Possible influencing factors were also analyzed. This review fills a current knowledge gap, improves our understanding of the potential hazards and ecological impacts of MPs in sedimentary environments, and provides a scientific basis and reference for subsequent research.

## 2. MPs in Sediments

**Figure 1:**
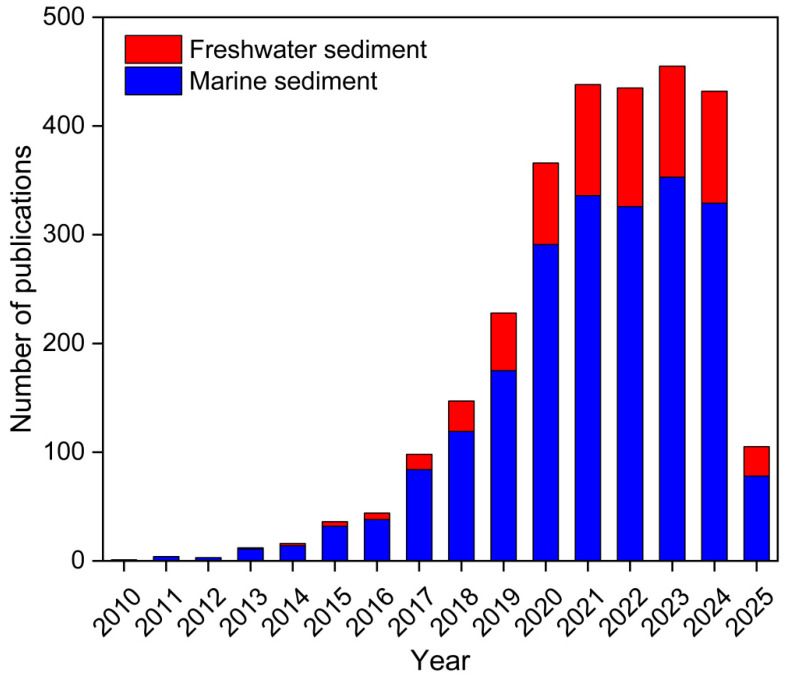
Numbers of publications on microplastics in marine and freshwater sediments.

Publications on MPs in sedimentary environments were statistically analyzed. The keywords “microplastic; freshwater; sediment” and “microplastic; marine; sediment” were used for search on Web of Science (https://www.webofscience.com/). As shown in Figure 1, MPs in marine and freshwater sediments have been drawing increasing levels of attention from scholars in recent years. MPs in marine sediments have been studied more extensively than those in freshwater sediments. Although research in recent years has focused on MPs in the ocean, freshwater systems (important sources and transport pathways for MPs entering oceans) are equally critical [20]. Freshwater environments (e.g., rivers and lakes) enrich plastic debris from urban runoff, sewage treatment plants and industrial emissions, ultimately conveying these plastic debris to the ocean [21]. Principal component analysis-multilinear regression analysis showed that land-based and marine sources of MPs in the whole Yellow Sea and Bohai Sea region accounted for 77% and 23%, respectively, of the total MP amount, with riverine inputs being the main source of MPs in the Bohai Sea [22]. This may enhance concentrations of MPs in both estuaries and oceans (especially in coastal areas), increasing the ecological risks of MPs.

**Figure 2:**
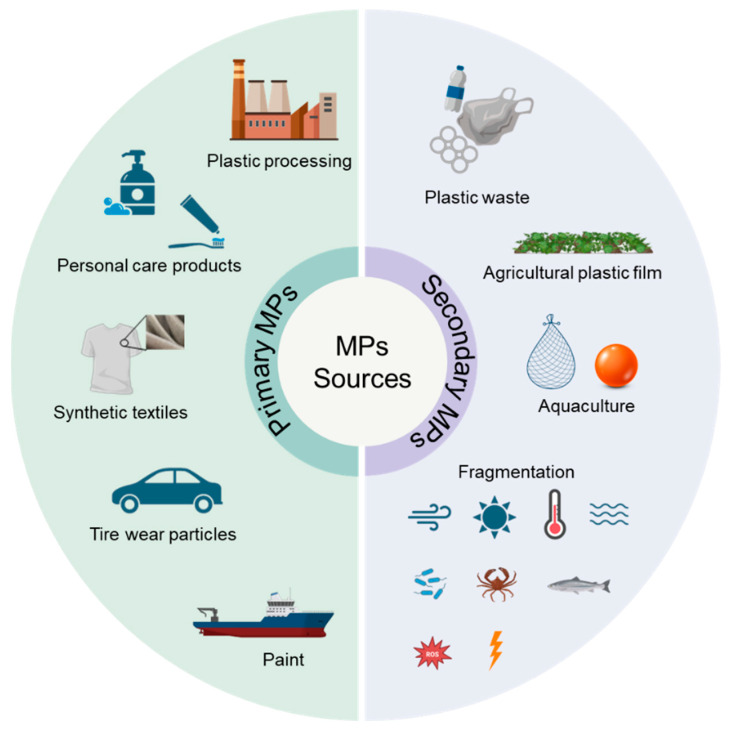
The major sources of microplastics in sediments.

The sources of MPs in aquatic environments can be divided into two major categories: primary MPs and secondary MPs (Figure 2). Primary MPs are intentionally synthesized at defined small sizes for commercial applications [23]. Primary sources mainly include plastic processing, personal care products (e.g., toothpastes and facial cleansers), synthetic textiles, tire wear particles, and paint [24,25,26]. Secondary MPs are formed by the fragmentation and decomposition of large items of plastic waste through physical, chemical, and biological progresses. Thompson et al. [4] demonstrated the significance of secondary MPs using the amount of leakage of macroplastics into the ocean (7.6 million tons per year). Secondary sources are mainly derived from plastic waste, agricultural plastic film, and aquaculture products [27,28,29]. These MPs entering the aquatic environment can reach sediments through sedimentation. Especially, biodegradable MPs (alternatives to conventional MPs) generally have a higher density than both seawater and freshwater (e.g., ρ_PLA_ = 1.26 g/cm^3^), causing them to be more inclined to settle into sediments [30]. The ecological responses of both conventional MPs and biodegradable MPs in sediments urgently need attention.

Regarding freshwater sediments, higher concentrations of MPs (up to thousands of particles/kg of sediment) have been found in sediments near to industrial areas [31] and sewage treatment plant outfalls [32]. Confluences of tributaries and main streams are prone to the accumulation of MPs due to the slowing down of water flow. Lentic environments such as lakes, reservoirs, bays, and other areas of slower flow are more prone to MP enrichment than lotic areas [33]. In terms of vertical distribution, surface sediments (0–5 cm) usually have the highest concentrations of MPs, which decrease with depth [34]. In freshwater sedimentary environments, MPs are most commonly found in fibrous form [35]. In general, MPs with particle diameters below 1 mm are more prevalent in freshwater sediments, and the abundance of MPs tends to decrease as particle size increases [36]. In the case of freshwater sediments, the distribution of MPs has been shown to exhibit a decreasing trend from nearshore to deep sea. In [37], ranking from highest to lowest, average concentrations of MPs were given for fjord (7000 particles kg^−1^ dry sediment), estuary (300 particles kg^−1^ dry sediment), beach (200 particles kg^−1^ dry sediment), shallow coast (200 particles kg^−1^ dry sediment), deep sea (80 particles kg^−1^ dry sediment), and continental shelf (50 particles kg^−1^ dry sediment). The MPs in marine sediments are mainly fibers and fragments, and the size is concentrated in a range of 0.1–1 mm [38].

## 3. Effects of MPs on Microbial Community in Sediments

### 3.1. The Colonization of Microorganisms on the Surface of MPs

**Figure 3:**
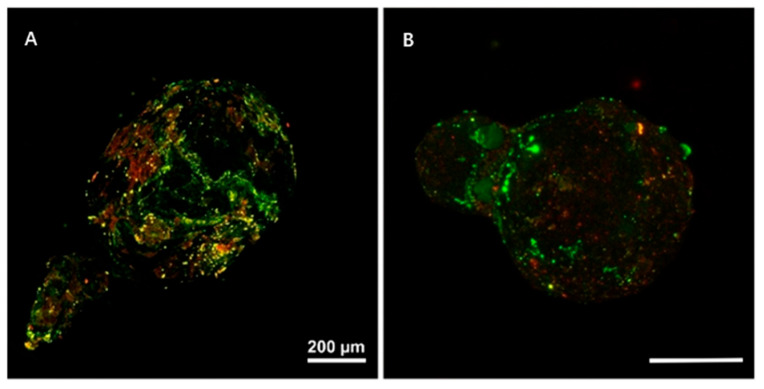
CLSM images of (**A**) PE and (**B**) PP MPs covered with biofilm. Red areas indicate colonization of bacteria, and green areas indicate EPS coverage. Reprinted with permission from reference [43].

In aquatic environments, the surface of plastic debris becomes a habitat for microbial communities, facilitating the formation of biofilms [39]. MPs can adsorb organic matter and nutrients from the surrounding environment, providing a favorable substrate for the colonization of microorganisms and serving as a suitable habitat for diverse bacterial communities [12]. The formation of biofilms on MP surfaces can be summarized into three stages: initial microbial attachment to the MPs surface, the formation of extracellular polymeric substances (EPSs), and subsequent microbial proliferation [40,41,42]. MPs covered with biofilms can be observed using confocal laser scanning microscopy (CLSM) (Figure 3). It was previously reported that colonization of microorganisms (red area) and coverage of EPS (green area) occurred on the surfaces of polyethylene (PE) and polypropylene (PP) MPs [43]. It has also been reported that bacterial communities on the surfaces of MPs exhibit higher stress resistance and greater ecological stability, compared with those in surface water and sediments [44].

The microorganisms forming biofilm on MPs primarily consist of algae, fungi, and bacteria, with bacteria being the most common [45]. For example, Neto et al. [46] reported that bacteria were the main colonizers on the surfaces of MPs in the sediments of Victoria Bay. In addition to bacteria, diatom residues, fungal filamentous bodies, and spores were also observed on MP surfaces. Among the identified bacteria taxa, *Proteobacteria* was the most common phylum. This may be attributed to the strong EPS secretion capacity of Proteobacteria (e.g., Pseudomonas), which facilitates their adhesion to MPs [47]. As Deng et al. [48] indicated, the dominant phylum on surfaces of PE MPs in the sediments of mangrove ecosystems is *Proteobacteria*. Furthermore, Ashar et al. [49] demonstrated that the most dominant bacterial phylum was *Proteobacteria* (>70%), and the most dominant fungi *Ascomycota* and *Basidiomycota*, on MP surfaces in marine and lake sediments.

**Figure 4:**
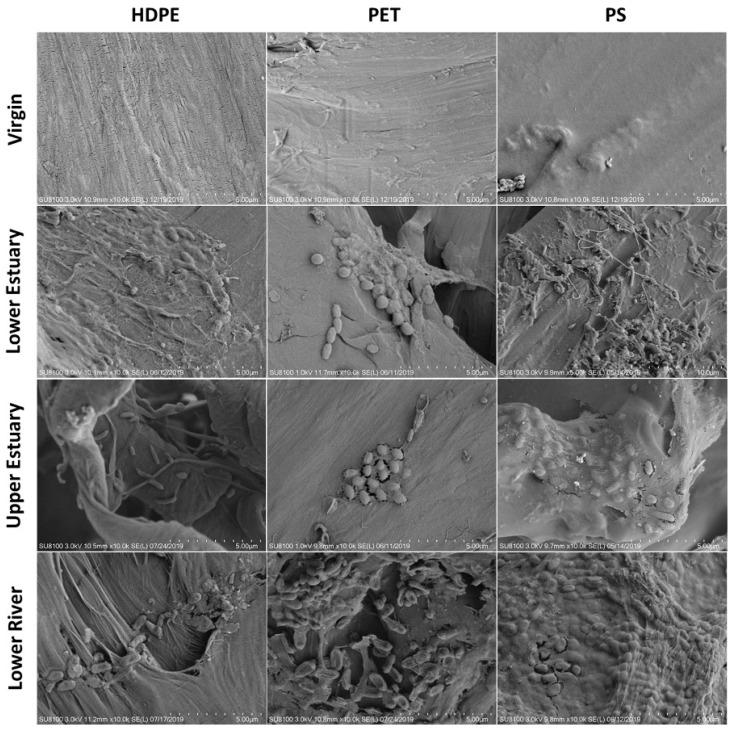
Scanning electron microscope (SEM) images of biofilm growth on the surfaces of HDPE, PET and PS MPs before and after 31 days in three different river areas. Reprinted with permission from reference [51].

The formation of biofilms on MPs is primarily affected by two major factors: the physicochemical properties of MPs, and environmental factors [50]. Qiang et al. [51] visually demonstrated that environmental factors (e.g., salinity) and MP types can affect the formation of biofilms and promote the colonization of different microbial communities in freshwater and estuary environments (Figure 4). A similar conclusion has also been reached with regard to sediments. Xie et al. [52] conducted exposure experiments involving nine types of MPs in the mangroves of Zhanjiang, revealing that different structures of MPs could lead to distinct microbial colonization on their surfaces. Rosato et al. [53] simulated the anaerobic ecosystem of marine sediments in Piallassa Baiona in 2020, and investigated the colonization of anaerobic bacterial communities on MPs such as polyethylene terephthalate (PET), polystyrene (PS), PP, PE and PVC. *Firmicutes* were dominant in the biofilm of MPs, while *Proteobacteria* accounted for only a small proportion. Subsequently, in 2022, microbial colonization on the same five types of MPs as mentioned above were studied in the anoxic marine sediments of Piallassa Baiona, revealing that *Proteobacteria* and *Firmicutes* were dominant on surfaces of all MPs [54]. Compared with the physicochemical properties of MPs, environmental factors have a greater impact on the formation of biofilms, possibly because environmental factors influence the entire formation process of biofilms [55,56,57].

### 3.2. Microbial Diversity and Dominant Bacterial Phyla Affected by MPs

Studies have indicated that MPs reduce the diversity of microorganisms in sediments [58]. This can be attributed to the direct toxicity of MPs on microorganisms or to the interference of MPs on the deposition environment [59,60]. Some studies have also shown that MPs increase the richness and diversity of microbial communities in sedimentary environments. Wang et al. [61] found that microbial abundance exhibited a positive correlation with MP content in a sedimentary environment. On the one hand, MPs promote the circulation of nutrients required for the growth and development of microorganisms. On the other hand, MPs provide good habitats for microbial communities [61]. Seeley et al. [18] demonstrated that PVC, polyurethane foam (PUF), and PLA increased microbial diversity in sediments, while PE decreased it. Li et al. [62] analyzed the α diversity of microbial communities in the sediments of the Huangjinxia Reservoir. The results showed that the α diversity of low-concentration MP treatments was higher than that of the high-concentration MP treatments, indicating that MP concentration is a key factor in influencing the diversity of microbials in the sedimentary environment. However, the influence of MP concentration on microbial communities is considerably less pronounced than that of MP type [63]. In addition, short-term exposure to MPs may increase diversity (providing new ecological niches) [64], and long-term exposure may lead to a decrease in diversity (accumulation of toxicity) [65]. Climate change (e.g., rising temperatures) drives changes in the sedimentary environment. It was reported that an increase in temperature intensified the negative impact of MPs on microbial communities (e.g., reducing microbial diversity) [66]. Furthermore, temperature can also cause differences in the responses of microbial activities (e.g., ammonia-oxidizing archaea) to MPs, ultimately affecting the nitrogen cycle process [67]. Taken together, the impacts of MPs on the diversity of microbials in sedimentary environments are related to the types of MPs involved and the nature of the sedimentary environment, as well as exposure time and exposure concentration.

Table 1 presents the effects of MPs on microbial abundances in sedimentary environments. It can be seen that the dominant bacterial phyla of the sediments in these studies all contain *Proteobacteria*, consistent with the finding mentioned in Section 3.1 that *Proteobacteria* were the dominant bacterial phyla on plastic rings. MPs can cause changes in the dominant bacterial phyla in sediments. For instance, Yin et al. [16] demonstrated that PVC and PLA increased the abundance of *Proteobacteria* in sediments, and PP enhanced abundances of the *Chloroflexi* and *Bacteroides* phyla. Zeng et al. [68] demonstrated that PET and PVC increased abundances of *Actinobacteriota* and *Proteobacteria* in sediment. Changes in the dominant phyla are key signals of functional transformation within the sediment microbial ecosystem. These shifts may trigger a cascade effect, affecting the entire process from microscopic metabolic activities to macroscopic ecological functions.

### 3.3. Effects of MPs on Microbial Functions

#### 3.3.1. Nitrification and Denitrification

Table 2 summarizes the impacts of MPs on sedimentary nitrification and denitrification. Discrepancies in results may be due to differences in the types of MPs and the sedimentary environments. It has been shown that MPs can interfere with nitrification and denitrification processes by disrupting related enzyme activities and the abundances of related functional genes encoding the enzymes [18,77]. It has also been shown that MPs can influence microbial functions by affecting the composition and structure of the microbial community. For example, Chen et al. [78] and Dai et al. [79] both showed that biofilms colonized on MP surfaces can reduce denitrifying bacteria and thus inhibit the denitrification process. Huang et al. [71] showed that PE MPs in sediment stimulated the growth of denitrifying bacteria and thus promoted the denitrification process. Other pathways of influence include the oxygen environment on the MP surface and the physicochemical properties of the sediment (e.g., porosity, nutrients, pH) [71,80,81]. Biofilm formation on plastic surfaces has been found to create anoxic conditions favorable for denitrifiers, facilitating the denitrification process and N_2_O production [47].

#### 3.3.2. Nitrogen Fixation

In contrast to conventional MPs, biodegradable MPs exhibit a significantly stronger promotion effect on nitrogen fixation in sediments. Yin et al. [16] found that biodegradable MPs increased the abundance of nitrogen-fixing enzymes and nitrogen-fixing genes (e.g., *nifD*, *nifH*, and *nifX*) in sediment microbes. This suggests that the presence of PLA MPs in sediments may induce microorganisms to show higher nitrogen fixation capacity. Sun et al. [86] also demonstrated that the nitrogen fixation function was higher in the biodegradable MP treatments, compared with conventional MPs, possibly due to the higher abundance of *Bradyrhizobiaceae* in biodegradable MP treatments. MPs may affect nitrogen fixation by altering the microbial community structure in sediments. It may be recalled from Section 3.2 that MPs can alter the abundance of *Proteobacteria* in sediment. In [87], it was shown that *Burkholderiaceae* were nitrogen-fixing bacteria in *Proteobacteria*.

## 4. Effects of MPs on Biogeochemical Cycling in Sedimentary Environments

**Figure 5:**
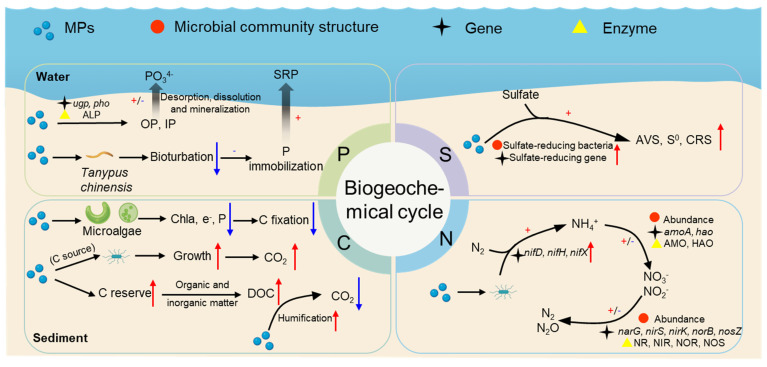
Influences and mechanisms of microplastics on carbon, nitrogen, phosphorus, and sulfur cycles in sediments. The red arrows indicate increase/promotion, and the blue arrows indicate decrease/inhibition.

Biogeochemical cycling refers to the process of cyclic flow of basic chemical elements between organisms and the external environment [88]. Microorganisms play a key driving role in biogeochemical cycling. Numerous studies have reported that MPs in sediments affect carbon, nitrogen, phosphorus and sulfur cycling through three primary mechanisms: influencing sediment microbial community diversity and structure; regulating the related enzyme activity; and modifying the abundance of genes encoding these enzymes. Other pathways (e.g., influencing the physicochemical properties of the sediment environment, interfering with benthic invertebrates) have been mentioned in a few studies. In this section, the influence mechanisms of MPs on carbon, nitrogen, phosphorus, and sulfur cycles in the sedimentary environment are reviewed based on available studies (Figure 5). In the 5, the red, black, and yellow symbols indicate that MPs regulate the biogeochemical cycle process by influencing the structure of microorganisms, gene abundance, and enzyme activity, respectively.

### 4.1. Nitrogen Cycle

As shown in the nitrogen-cycle section of Figure 5, MPs can influence nitrogen fixation processes by regulating nitrogen fixation-related genes of sediment microbes. Yin et al. [16] showed that biodegradable MPs can increase abundances of *nifD*, *nifH*, and *nifX* genes in sediment microorganisms, resulting in a higher nitrogen fixation potential. Additionally, MPs also affect the nitrification and denitrification processes through different pathways. Huang et al. [71] showed that PE MPs in sediments stimulated the growth of denitrifying bacteria and anaerobic ammonia-oxidizing bacteria, thus facilitating denitrification. Additionally, Yin et al. [16] showed that PP MP-treated sediment had the highest relative abundance of *Nitrospiaceae*, while the lowest abundance of *Nitrospiaceae* was found in their PLA MP-treated group. It was also explained that conventional PP MPs boosted nitrification, whereas biodegradable MPs inhibited nitrification. The above studies suggest that MPs affect the nitrification and denitrification process by changing the dominant species in the sedimentary environment and the relative abundances of certain functional microorganisms involved in nitrogen metabolism. Additionally, MPs can disrupt the nitrification and denitrification process by lowering the activity of related enzymes (e.g., ammonia monooxygenase (AMO), hydroxylamine oxidoreductase (HAO), nitrate reductase (NR), nitrite reductase (NIR), nitric oxide reductase (NOR), and nitrous oxide reductase (NOS)), along with abundances of relevant functional genes encoding the enzymes (e.g., *amoA*, *hao*, *narG*, *nirS*, *nirK*, *norB*, *nosZ*) [18,77]. For instance, Seeley et al. [18] experimentally found the lowest abundance of *amoA* genes (ammonia oxidation-related enzyme genes) in their PVC-treated group, indicating that PVC treatment inhibited nitrification.

### 4.2. Carbon Cycle

The mechanisms by which MPs affect the carbon cycle in sedimentary environments are shown in Figure 5. The presence of MP can increase the carbon stock and affect the carbon cycle in the sedimentary environment. MPs (especially biodegradable MPs) and their degradation intermediates can serve as a carbon source for microbial life metabolism [88]. Chen et al. [76] showed that degradable MPs was more readily biodegradable, thus providing more carbon sources and promoting microbial growth. In addition, MPs are capable of absorbing organic carbon from the surrounding environment, thus influencing carbon storage in sediments [89,90]. Plastic debris entering the ocean can react with organic and inorganic matter, which may promote the release of surface microlayer dissolved organic carbon (DOC), thus increasing carbon storage in the ecosystem [91]. Liu et al. found an increase in total organic carbon (TOC) in sediments with added MPs, which further confirmed that MPs make a significant contribution to organic carbon levels in sediments [92]. MPs in sediments can promote the humification of DOC and thus inhibit the release of CO_2_ [93].

MPs can adversely affect carbon fixation in the ocean [94]. In sedimentary environments, algae, and submerged plants are crucial in carbon fixation. Wang et al. [95] showed that MPs can reduce chlorophyll content in freshwater microalgae cells, thus decreasing the carbon fixation capacity of microalgae. You et al. [96] showed that MPs can inhibit electron transport and photosynthetic efficiency in cyanobacteria, leading to inactivation of carbon fixation enzymes. Additionally, Ding et al. [83] found that tire particles (TPs) were generally able to inhibit carbon fixation in the sediment environment, as revealed by analysis of functional genes, although TP exposure led to the enrichment of bacteria associated with hydrocarbon degradation. Recent studies have also clarified the combined effects of MPs and co-existing pollutants on microorganisms and carbon cycles in sediments. Liu et al. [92] found that a combined exposure of MPs and decabrominated diphenyl ether (deca-BDE) had synergistic effects on microbial diversity, community structure and functions. For instance, co-exposure increased abundances of hydrogenotrophic methanogenesis and methylotrophic methanogenesis, promoting the carbon cycle in sediments [92].

### 4.3. Phosphorus Cycling

As shown in Figure 5, phosphorus in sediments is derived from nutrient sinks and also plays a vital role in biogeochemical processes [69,97]. The phosphorus cycle in sedimentary environments is mainly driven by organophosphorus mineralizing and inorganic phosphorus-dissolving bacteria. These bacteria contribute to the phosphorus cycle by secreting series of extracellular enzymes that facilitate inorganic phosphorus conversion and organic phosphorus mineralization [98,99,100]. Studies have demonstrated that phosphorus stored in sediments can enter the aqueous phase via desorption, dissolution, and mineralization processes [101]. MPs can affect the release of phosphorus from sediments, thus altering the phosphorus content in sediments. For example, Yin et al. [16] showed experimentally that PP and PLA MPs promoted the release of phosphorus from sediments into overlying water, while PVC inhibited the release of phosphorus from sediments.

MPs can affect phosphorus cycling by regulating enzymes, genes, and proteins related to organic phosphorus mineralization and inorganic phosphorus conversion. For example, Yin et al. [16] indicated that PP MPs significantly increased alkaline phosphatase (ALP) activity, while PLA MPs significantly decreased ALP activity. PP MPs significantly increased phosphorus regulatory (*phoR*) gene abundance, while PLA MPs significantly down-regulated microbial organophosphorus mineralization (*phoD*) genes and phosphorus regulatory (*phoB* and *phoR*) genes abundance. It can be assumed that PLA MPs inhibited the conversion of organic phosphorus to soluble inorganic phosphorus by reducing ALP activity and down-regulating *phoD*, *phoB*, and *phoR* genes. In addition, abundances of phosphorus transport proteins (*ugpA*, *ugpB*, *ugpC*, and *ugpE*) genes in the sediment also exhibited differences, with significantly lower abundances in PP treatments than in the PLA treatments [16]. In contrast, Ding et al. [83] showed that high doses of TP were able to increase the activities of most target enzymes associated with phosphorus cycling, but the functional genes associated with phosphorus cycling showed a decreasing trend.

The physiological activities of benthic invertebrates can alter the sediment matrix, thereby influencing nutrient cycle. Song et al. [102] demonstrated that MPs caused biological disturbance in *Tanypus chinensis*, leading to hypoxia in the sediment. Anoxic conditions are conducive to the reduction of Fe^3+^ in sediments; in addition, they facilitate the release of Fe-P into pore water, further leading to limited immobilization of sedimentary phosphorus and a high level of soluble reactive phosphorus (SRP) content in the water.

### 4.4. Sulfur Cycling

Compared with the influence of MPs on carbon and nitrogen cycles in the sedimentary environment, little is known about their influence on sulfur cycles. The sulfur cycle is interrelated with other material cycling processes (e.g., the carbon cycle and the nitrogen cycle), jointly promoting material circulation and energy flow and maintaining the stability of the ecosystem [103]. Recent studies have largely focused on the impacts of MPs on sulfur cycling in coastal-zone sedimentary environments. Mangrove wetlands are one of the most important ecosystems in coastal zones, being characterized by high sulfate content, high numbers of sulfide species, and active nutrient cycling driven by microorganisms. *Proteobacteria*, *Firmicutes*, and *Chloroflexia* are considered to be the main bacteria involved in the sulfur cycle [104]. A variety of sulfur oxidation- or reduction-related microorganisms drive the sulfur cycle. Investigating the effects of MPs on these microorganisms is crucial for maintaining a safe and stable ecosystem.

The known mechanisms by which MPs affect the sulfur cycle in sediments are illustrated in Figure 5 above. Wang et al. [19] used sulfur stable isotope analysis to demonstrate that PLA MPs can contribute to the sulfur cycle in mangrove sediments by facilitating the reduction of sulfate to acid volatile sulfide (AVS), singlet sulfur (S^0^), and chromium-reducible S (CRS). This study further employed macro genome sequencing techniques to analyze sulfur cycle-related bacteria and genes, revealing that PLA MPs could increase abundances of sulfate-reducing bacteria and sulfate-reducing genes [19]. In addition, Ding et al. [83] showed that TP could reduce abundances of sulfur cycle-related genes. It has been speculated that PLA MPs are more conducive to degradation by microorganisms, providing more carbon sources and thus promoting microbial growth. The above studies suggest that MPs can influence the structure of sediment microbial communities and related functional genes, and then affect the sulfur cycle. Additionally, Pinnell and Turner [97] showed that bioplastics can attach in significant quantities to sulfate and sulfite reductase, as well as to sulfate-reducing microorganisms in the benthic environment which have a more serious impact on biogeochemical processes.

## 5. Conclusions and Outlook

This review summarizes studies on the impacts of MPs on colonization, diversity, structure, and function in sedimentary microbial communities. The impacts of MPs on microbial communities are influenced by multiple factors, such as sediment and MP types, which can lead to variable outcomes. Compared with conventional MPs, biodegradable MPs have more significant effects on microbial community structures and functions because of their easy biodegradation and utilization. MPs seriously disrupt biogeochemical cycles in sedimentary environments, primarily by altering microbial community structures, activities of related enzymes, and abundances of genes involved in the cycles of carbon, nitrogen, phosphorus, and sulfur. Investigating the underlying mechanisms is essential to assess and maintain ecosystem security and stability.

Although there have been numerous studies explaining the mechanisms of MP-microbial community interaction in sediments, a number of knowledge gaps may be suggested, as follows:

(1) Because research content is trivial and the influencing factors are complex, it is challenging to establish a universal and common law. Discrepancies in the effects of environmental factors and different MP types on microbial communities still need to be further explored.
(2) Current research is still focused on the short-term effect of MPs on microorganisms; however, the aging of MPs will lead to large changes in their properties. The attachment of biofilm can enhance their electronegativity. In addition, the photoaging of MPS increases numbers of oxygen-containing functional groups on the polymer molecular chain and also increases hydrophilicity. The physicochemical properties of MPs undergo significant changes over time, leading to alterations in their environmental behavior and biological toxicity in aquatic and sedimentary environments. These dynamic transformations are worth exploring further in future research. The following summarizes methods of research on MP aging:
  - Laboratory-based aging simulations can be used to simulate natural environmental conditions over extended periods (e.g., UV radiation, temperature fluctuations and eco-corona).
  - Long-term in situ aging of MPs is carried out in a real environment (e.g., marine and freshwater sediments).
(3) There is a relative lack of research on the effects of MPs on biogeochemical cycles in sedimentary environments, especially phosphorus and sulfur cycles. Therefore, further studies are necessary to elucidate the response of elemental cycles after MP pollution in sedimentary environments, and to assess the broader implications for aquatic ecosystems and human health. Research gaps with regard to phosphorus and sulfur cycles might be attributed to an absence of standardized indicators. Future research can focus on the following crucial microbiota relevant to phosphorus and sulfur cycles: phosphate-solubilizing bacteria (PSB); phosphate-accumulating organisms (PAOs); sulfate-reducing bacteria (SRB); sulfur-oxidizing bacteria (SOB); and sulfur-disproportionating bacteria.

## Figures and Tables

**Table 1:** Impacts of microplastics on microbial community structures in sediments.

MPs	Dominant Species	Abundance		Reference
		Decreasing	Increasing	
PE, PVC, PUF, PLA	*Bacteriodes*, *Proteobacteria* (classes *Deltaproteobacteria* and *Gammaproteobacteria*)	PVC: *Chromatiaceae*, *Ectothiorhodospiraceae*, *Lentimicrobiaceae*, *Magnetococcaceae*, *Pirellulaceae*, *Sedimenticolaceae*, *Thermoanaerobaculaceae*, *Woeseiaceae*.	All: *Family_XII* PE, PUF, PVC: *Izimaplasmataceae*, *Marinifilaceae*, *Marinilabiliaceae* PVC: *Desulfobacterace*, *Desulfobulbaceae*, *Acholeplasmataceae*, *Anaerolineaceae*, *Family_XII*, *Izimaplasmataceae*, *Lachnospiraceae* and *Marinilabiliaceae*	[18]
PVC, PP, PLA	*Proteobacteria*, *Acidobacteriota*, *Chloroflexi*, *Bacteroidota*, *Desulfobacterota*	PP, PLA: *Actinobacteriota*, *Nitrospirota* PP, PVC: *Pseudomonadaceae*	PVC, PLA: *Proteobacteria*, *Firmicutes* PP: *Chloroflexi*, *Bacteroidota*, *Myxococcota* PLA: *Anaerovoracaceae*, *Burkholderiaceae*, *Chromobacteriaceae*, *Desulfocapsaceae*, *Geobacteraceae*, *OPB41*, *Pseudomonadaceae*, *Syntrophomonadaceae* PP: *Bacteroidetes_vadinHA17*, *BSV26*, *Gallionellaceae*, *Hydrogenophilaceae*, *Lentimicrobiaceae*, *Nitrospiraceae*, *Nitrosomonadaceae*, *Prolixibacteraceae*, *Saprospiraceae*, and *Spirochaetaceae*	[16]
PE, PET, PVC	*Proteobacteria*, *Actinobacteriota*	PET: *Sva0081 sediment group*	All: *Proteobacteria*, *Actinobacteriota*(*except in PE*) PET, PVC: *Woeseia*, *Subgroup 10* PVC: *Halioglobus*, *Sulfurovum*, *Ilumatobacter*	[68]
TWPs	*Proteobacteria*, *Desulfobacteriota*		Defluvimonas, Defluviitaleaceae, Fusibacter, Lutibacter, Desulfopila, Citrobacter, Stenotrophomonas	[69]
	*Proteobacteria*, *Bacteroidetes*	The abundance of MPs positively correlated with *Vibrio*, *Pseudomonas*, *Subgroup-10*, *Bacillus*, *Streptococcus*, *Flavisolibacter*, *Cm1-21* The abundance of MPs negatively correlated with *Sphingomonas*		[70]
PE	*Proteobacteria*, *Firmicutes*, *Chloroflexi*	The concentration of MPs negatively correlated with *Proteobacteria* The concentration of MPs positively correlated with *Bacteroidetes*		[71]
PE			*Actinobacteria*, *Firmicutes*	[72]
PVC			*Bacteria (Actinobacteria*, *Bacteroidetes*, *Chloroflexi*, *Firmicutes*, *Gemmatimonadetes*, *and Planctomycetes) and eukaryotes (Ascomycota*, *Bacillariophyta*, *Chordata*, *and Streptophyta)*	[73]
PE, PVC	*Chloroflexi*, *Proteobacteria*, *Bacteroidota*, *Firmicutes*, *Actinobacteriota*		*Proteobacteria*, *Bacteroidota*	[14]
PE, PS, PVC, PLA	*Proteobacteria (Betaproteobacteria and Gammaproteobacteria)*, *Bacteroides*, *Nitrospirae*	All: *Bacteroidales*, *Sinobacteraceae*, *Saprospiraceae*, *Alcaligenaceae*, *Syntrophaceae* and *Rhodocyclace* PS: *Bacteroidales*	PVC: *Comamonadaceae*, *Clostridiales* and *Myxococcales*	[74]
PET	*Proteobacteria*, *Chloroflexi*, *Actinbacteriota*, *Bacterioidota*, *Desulfobacterota*, *Acidobacteriota*	*Acidiferrobacteraceae*, *Cellulomonadaceae*, *norank_o_EPR3968-O8a-Bc78*, *norank_o__Oligoflexales*, *Vulgatibacteraceae*	*FTLpost3*, *MAT_CR_H6_H10*, *MWH-UniP_aquatic_group*, *norank_o_Chthonomonadal*, *norank_o__norank_c__Microgenomatia*, *Pedosphaeraceae*, *Saprospiraceae*	[75]
PE, PLA, PVC	*Bacteroidota*, *Proteobacteria*, *Firmicutes*, *Actinobacteria*, *Chloroflexi*, *Acidobacteriota*		PLA: *Nitrospria*, *Bacillus*, *Pseudomonas*, *Paracoccus*, *Acinetobacter* PVC: *Nitrospira* and *Nitrosomonas* PE: *Peudomonas*, *Paracoccus*, *Acinetobacter*, *Flavobacterium*, *Nocardia*, *Thiobacillus*, *Arthrobacter*, *Azoarcus*, *Rhodococcus* PLA and/or PE: *Bacillus*, *Pseudomonas* and *Nocardia*	[76]
PE, PLA, PET, PBS, PC	*Proteobacteria*, *Chloroflexi*, *Actinobacteriota*	PLA: *Thermodesulfovibrionia*, *Desulfobacteria*, *Vicinamibac teria*, *Thermoleophilia*, *Acidimicrobiia* and *KD4–96*	PLA: *Gamma-proteobacteria*, *Clostridia*, *Negativicutes* and *Cam pylobacteria*	[58]

**Table 2:** Impacts of microplastics on nitrification and denitrification in sedimentary environments. “+” indicates promotion, “−” indicates inhibition.

Location	Microplastic	Nitrification	Denitrification	Reference
Blue Sea Aquaculture Farm, Shanghai, China	PA	(+)		[82]
The Bohai Sea	PE, PET, PVC	PE and PET (+); PVC (−)	PVC (+)	[68]
The York River estuary in Gloucester Point, VA	PVC, PUF, PLA	PVC (−); PUF and PLA (+)	PVC (−); PUF and PLA (+)	[18]
Lake Mati	PET	(+)	(+)	[75]
Gaoyang Lake	PLA, PVC, PP	PLA (−); PVC, PP (+)	PLA (+)	[16]
Low tide of an intertidal marsh in Yantai, China	TP	(−)		[83]
Chongming eastern intertidal flat	PLA		PLA (+)	[76]
The Daihai Lake in Ulaanchab, Inner Mongolia Autonomous Region	PE, PVC		(−)	[14]
Lake Jinhu in Chongqing, China	PE		(+)	[71]
Nearshore area of Liaohe Estuary National Nature Reserve, China	TWP		(+)	[69]
29°04–29°22 N, 112°17–112°42 E	PE	(+)		[72]
Yangchun Lake, Wuhan, China	PLA, PBAT		(−)	[84]
Suishi Ferry of the Pearl River, Guangzhou, China	PE, PS, PLA, PVC	(−)	(−)	[74]
Core mature mangrove area in Shantou City, southeastern China	PE, PLA, PET, PBS, PC	PC (−); PE (+); PLA (+)	PLA (+); PC (−); PE (+)	[58]
Poyang Lake wetland in China			(+)	[85]

## References

[1] Shen M., Song B., Zeng G., Zhang Y., Huang W., Wen X., Tang W. (2020). Are biodegradable plastics a promising solution to solve the global plastic pollution?. Environ. Pollut..

[2] Mao R., Lang M., Yu X., Wu R., Yang X., Guo X. (2020). Aging mechanism of microplastics with UV irradiation and its effects on the adsorption of heavy metals. J. Hazard. Mater..

[3] Hodgson D.J., Bréchon A.L., Thompson R.C. (2018). Ingestion and fragmentation of plastic carrier bags by the amphipod Orchestia gammarellus: Effects of plastic type and fouling load. Mar. Pollut. Bull..

[4] Thompson R.C., Courtene-Jones W., Boucher J., Pahl S., Raubenheimer K., Koelmans A.A. (2024). Twenty years of microplastic pollution research—What have we learned?. Science.

[5] Cao J., Yang Q., Jiang J., Dalu T., Kadushkin A., Singh J., Fakhrullin R., Wang F., Cai X., Li R. (2022). Coronas of micro/nano plastics: A key determinant in their risk assessments. Part. Fibre Toxicol..

[6] Eriksen M., Lebreton L.C., Carson H.S., Thiel M., Moore C.J., Borerro J.C., Galgani F., Ryan P.G., Reisser J. (2014). Plastic pollution in the world’s oceans: More than 5 trillion plastic pieces weighing over 250,000 tons afloat at sea. PLoS ONE.

[7] Boucher C., Morin M., Bendell L.I. (2016). The influence of cosmetic microbeads on the sorptive behavior of cadmium and lead within intertidal sediments: A laboratory study. Reg. Stud. Mar. Sci..

[8] Kuypers M.M., Marchant H.K., Kartal B. (2018). The microbial nitrogen-cycling network. Nat. Rev. Microbiol..

[9] Wu H., Cui H., Fu C., Li R., Qi F., Liu Z., Yang G., Xiao K., Qiao M. (2024). Unveiling the crucial role of soil microorganisms in carbon cycling: A review. Sci. Total Environ..

[10] Wang F., Bai Y., Yang F., Zhu Q., Zhao Q., Zhang X., Wei Y., Liao H. (2021). Degradation of nitrogen, phosphorus, and organic matter in urban river sediments by adding microorganisms. Sustainability.

[11] Vijayanand M., Ramakrishnan A., Subramanian R., Issac P.K., Nasr M., Khoo K.S., Rajagopal R., Greff B., Azelee N.I.W., Jeon B. (2023). Polyaromatic hydrocarbons (PAHs) in the water environment: A review on toxicity, microbial biodegradation, systematic biological advancements, and environmental fate. Environ. Res..

[12] Shen M., Zhu Y., Zhang Y., Zeng G., Wen X., Yi H., Ye S., Ren X., Song B. (2019). Micro (nano) plastics: Unignorable vectors for organisms. Mar. Pollut. Bull..

[13] Song Q., Nie F., Zhu D., Wei Y., Zhang M., Hu Y., Chen M., Kang D., Chen Z., Lin H. (2022). Microplastic pollution and its impact on marine microbes in Zhanjiang, China. J. Coast. Conserv..

[14] Li W., Wang Z., Li W., Li Z. (2022). Impacts of microplastics addition on sediment environmental properties, enzymatic activities and bacterial diversity. Chemosphere.

[15] Yu Y., Li X., Feng Z., Xiao M., Ge T., Li Y., Yao H. (2022). Polyethylene microplastics alter the microbial functional gene abundances and increase nitrous oxide emissions from paddy soils. J. Hazard. Mater..

[16] Yin M., Yan B., Wang H., Wu Y., Wang X., Wang J., Zhu Z., Yan X., Liu Y., Liu M. (2023). Effects of microplastics on nitrogen and phosphorus cycles and microbial communities in sediments. Environ. Pollut..

[17] Wang X., Li J., Wang D., Sun C., Zhang X., Zhao J., Teng J., Wang Q. (2024). Unveiling microplastic’s role in nitrogen cycling: Metagenomic insights from estuarine sediment microcosms. Environ. Pollut..

[18] Seeley M.E., Song B., Passie R., Hale R.C. (2020). Microplastics affect sedimentary microbial communities and nitrogen cycling. Nat. Commun..

[19] Wang H., Yang Q., Li D., Wu J., Yang S., Deng Y., Luo C., Jia W., Zhong Y., Peng P. (2023). Stable isotopic and metagenomic analyses reveal microbial-mediated effects of microplastics on sulfur cycling in coastal sediments. Environ. Sci. Technol..

[20] Li J., Liu H., Chen J.P. (2018). Microplastics in freshwater systems: A review on occurrence, environmental effects, and methods for microplastics detection. Water Res..

[21] Lin L., Zuo L., Peng J., Cai L., Fok L., Yan Y., Li H., Xu X. (2018). Occurrence and distribution of microplastics in an urban river: A case study in the Pearl River along Guangzhou City, China. Sci. Total Environ..

[22] Zhang M., Lin Y., Booth A.M., Song X., Cui Y., Xia B., Gu Z., Li Y., Liu F., Cai M. (2022). Fate, source and mass budget of sedimentary microplastics in the Bohai Sea and the Yellow Sea. Environ. Pollut..

[23] Xu C., Zhang B., Gu C., Shen C., Yin S., Aamir M., Li F. (2020). Are we underestimating the sources of microplastic pollution in terrestrial environment?. J. Hazard. Mater..

[24] Hu Z., Sun Y., Zhou J., Sun W., Shah K.J. (2024). Microplastics in wastewater plants: A review of sources, characteristics, distribution and removal technologies. J. Contam. Hydrol..

[25] Ryberg M.W., Hauschild M.Z., Wang F., Averous-Monnery S., Laurent A. (2019). Global environmental losses of plastics across their value chains. Resour. Conserv. Recycl..

[26] Knight L.J., Parker-Jurd F.N., Al-Sid-Cheikh M., Thompson R.C. (2020). Tyre wear particles: An abundant yet widely unreported microplastic?. Environ. Sci. Pollut. Res..

[27] Belioka M., Achilias D.S. (2024). The effect of weathering conditions in combination with natural phenomena/disasters on microplastics’ transport from aquatic environments to agricultural soils. Microplastics.

[28] Song Y.K., Kim T., Shim W.J., Hong S.H., Im D. (2025). Microplastic emissions from fishing ropes: Quantification, characteristics, and implications for marine pollution. Mar. Pollut. Bull..

[29] Yu F., Pei Y., Zhang X., Wu X., Zhang G., Ma J. (2023). Occurrence and distribution characteristics of aged microplastics in the surface water, sediment, and crabs of the aquaculture pond in the Yangtze River Delta of China. Sci. Total Environ..

[30] Shi C., Zhang Y., Shao Y., Ray S.S., Wang B., Zhao Z., Yu B., Zhang X., Li W., Ding J. (2024). A review on the occurrence, detection methods, and ecotoxicity of biodegradable microplastics in the aquatic environment: New cause for concern. TrAC Trends Anal. Chem..

[31] Dhivert E., Pruvost J., Winiarski T., Gasperi J., Delor-Jestin F., Tassin B., Mourier B. (2024). Time-varying microplastic contributions of a large urban and industrial area to river sediments. Environ. Pollut..

[32] Margenat H., Nel H.A., Stonedahl S.H., Krause S., Sabater F., Drummond J.D. (2021). Hydrologic controls on the accumulation of different sized microplastics in the streambed sediments downstream of a wastewater treatment plant (*Catalonia, Spain*). Environ. Res. Lett..

[33] Mehra S., Sharma K., Sharma G., Singh M., Chadha P., Nuro A. (2020). Sources, fate, and impact of microplastics in aquatic environment. Emerging Contaminants.

[34] Yuan B., Gan W., Sun J., Lin B., Chen Z. (2023). Depth profiles of microplastics in sediments from inland water to coast and their influential factors. Sci. Total Environ..

[35] Yang L., Zhang Y., Kang S., Wang Z., Wu C. (2021). Microplastics in freshwater sediment: A review on methods, occurrence, and sources. Sci. Total Environ..

[36] Corcoran P.L., Norris T., Ceccanese T., Walzak M.J., Helm P.A., Marvin C.H. (2015). Hidden plastics of Lake Ontario, Canada and their potential preservation in the sediment record. Environ. Pollut..

[37] Harris P.T. (2020). The fate of microplastic in marine sedimentary environments: A review and synthesis. Mar. Pollut. Bull..

[38] Bouzekry A., Mghili B., Mancuso M., Bouadil O., Bottari T., Aksissou M. (2024). Anthropogenic Microparticles Abundance in Sandy Beach Sediments along the Tetouan Coast (Morocco Mediterranean). Environments.

[39] Guo W., Li D., Chen B., Li J., Li Z., Cao X., Qiu H., Zhao L. (2025). Microbial colonization on four types of microplastics to form biofilm differentially affecting organic contaminant biodegradation. Chem. Eng. J..

[40] Zettler E.R., Mincer T.J., Amaral-Zettler L.A. (2013). Life in the “plastisphere”: Microbial communities on plastic marine debris. Environ. Sci. Technol..

[41] Nauendorf A., Krause S., Bigalke N.K., Gorb E.V., Gorb S.N., Haeckel M., Wahl M., Treude T. (2016). Microbial colonization and degradation of polyethylene and biodegradable plastic bags in temperate fine-grained organic-rich marine sediments. Mar. Pollut. Bull..

[42] Li Y., Yang R., Guo L., Gao W., Su P., Xu Z., Xiao H., Ma Z., Liu X., Gao P. (2022). The composition, biotic network, and assembly of plastisphere protistan taxonomic and functional communities in plastic-mulching croplands. J. Hazard. Mater..

[43] Sturm M.T., Schuhen K., Horn H. (2022). Method for rapid biofilm cultivation on microplastics and investigation of its effect on the agglomeration and removal of microplastics using organosilanes. Sci. Total Environ..

[44] Wu N., Zhang Y., Zhao Z., He J., Li W., Li J., Xu W., Ma Y., Niu Z. (2020). Colonization characteristics of bacterial communities on microplastics compared with ambient environments (water and sediment) in Haihe Estuary. Sci. Total Environ..

[45] Wang J., Guo X., Xue J. (2021). Biofilm-developed microplastics as vectors of pollutants in aquatic environments. Environ. Sci. Technol..

[46] Neto J.A.B., Gaylarde C., Beech I., Bastos A.C., Da Silva Quaresma V., de Carvalho D.G. (2019). Microplastics and attached microorganisms in sediments of the Vitória bay estuarine system in SE Brazil. Ocean Coast. Manag..

[47] Su X., Yang L., Yang K., Tang Y., Wen T., Wang Y., Rillig M.C., Rohe L., Pan J., Li H. (2022). Estuarine plastisphere as an overlooked source of N_2_O production. Nat. Commun..

[48] Deng H., Fu Q., Zhang Y., Li D., He J., Feng D., Zhao Y., Yu H., Ge C. (2022). Bacterial communities on polyethylene microplastics in mangrove ecosystems as a function of exposure sites: Compositions and ecological functions. J. Environ. Chem. Eng..

[49] Ashar M., Fraser M.A., Li J., Wang C., Huang W., Zhang D., Zhang C. (2020). Interaction between microbial communities and various plastic types under different aquatic systems. Mar. Environ. Res..

[50] Carson H.S., Nerheim M.S., Carroll K.A., Eriksen M. (2013). The plastic-associated microorganisms of the North Pacific Gyre. Mar. Pollut. Bull..

[51] Qiang L., Cheng J., Mirzoyan S., Kerkhof L.J., Häggblom M.M. (2021). Characterization of microplastic-associated biofilm development along a freshwater-estuarine gradient. Environ. Sci. Technol..

[52] Xie H., Chen J., Feng L., He L., Zhou C., Hong P., Sun S., Zhao H., Liang Y., Ren L. (2021). Chemotaxis-selective colonization of mangrove rhizosphere microbes on nine different microplastics. Sci. Total Environ..

[53] Rosato A., Barone M., Negroni A., Brigidi P., Fava F., Xu P., Candela M., Zanaroli G. (2020). Microbial colonization of different microplastic types and biotransformation of sorbed PCBs by a marine anaerobic bacterial community. Sci. Total Environ..

[54] Rosato A., Barone M., Negroni A., Brigidi P., Fava F., Biagi E., Candela M., Zanaroli G. (2022). Bacterial colonization dynamics of different microplastic types in an anoxic salt marsh sediment and impact of adsorbed polychlorinated biphenyls on the plastisphere. Environ. Pollut..

[55] Li W., Zhang Y., Wu N., Zhao Z., Xu W.A., Ma Y., Niu Z. (2019). Colonization characteristics of bacterial communities on plastic debris influenced by environmental factors and polymer types in the Haihe Estuary of Bohai Bay, China. Environ. Sci. Technol..

[56] Miao L., Yu Y., Adyel T.M., Wang C., Liu Z., Liu S., Huang L., You G., Meng M., Qu H. (2021). Distinct microbial metabolic activities of biofilms colonizing microplastics in three freshwater ecosystems. J. Hazard. Mater..

[57] Xu X., Wang S., Gao F., Li J., Zheng L., Sun C., He C., Wang Z., Qu L. (2019). Marine microplastic-associated bacterial community succession in response to geography, exposure time, and plastic type in China’s coastal seawaters. Mar. Pollut. Bull..

[58] Fang C., Yang Y., Zhang S., He Y., Pan S., Zhou L., Wang J., Yang H. (2024). Unveiling the impact of microplastics with distinct polymer types and concentrations on tidal sediment microbiome and nitrogen cycling. J. Hazard. Mater..

[59] Hao Y., Sun Y., Li M., Fang X., Wang Z., Zuo J., Zhang C. (2023). Adverse effects of polystyrene microplastics in the freshwater commercial fish, grass carp (*Ctenopharyngodon idella*): Emphasis on physiological response and intestinal microbiome. Sci. Total Environ..

[60] Fang C., He Y., Yang Y., Fu B., Pan S., Jiao F., Wang J., Yang H. (2023). Laboratory tidal microcosm deciphers responses of sediment archaeal and bacterial communities to microplastic exposure. J. Hazard. Mater..

[61] Wang Y., Zhang G., Zhang F., Wang H. (2023). Diagnostic strategy for the combined effects of microplastics and potentially toxic elements on microbial communities in catchment scale. Sci. Total Environ..

[62] Li C., Gan Y., Dong J., Fang J., Chen H., Quan Q., Liu J. (2020). Impact of microplastics on microbial community in sediments of the Huangjinxia Reservoir-water source of a water diversion project in western China. Chemosphere.

[63] Chi J., Zhang H., Zhao D. (2021). Impact of microplastic addition on degradation of dibutyl phthalate in offshore sediments. Mar. Pollut. Bull..

[64] Kleinteich J., Seidensticker S., Marggrander N., Zarfl C. (2018). Microplastics reduce short-term effects of environmental contaminants. Part II: Polyethylene particles decrease the effect of polycyclic aromatic hydrocarbons on microorganisms. Int. J. Environ. Res. Public Health.

[65] Deng W., Zhang X., Liu W., Wang X., Wang Z., Liu J., Zhai W., Wang J., Zhao Z. (2025). Deciphering the effects of long-term exposure to conventional and biodegradable microplastics on the soil microbiome. J. Hazard. Mater..

[66] Wang Z., Xie J., Wang G., Yang H., Li Z., Zhang K., Shu R., Xie W., Tian J., Li H. (2025). Enhanced gut damage and microbial imbalance in bullfrog tadpoles (*Lithobates catesbeiana*) exposed to polystyrene microplastics under high-temperature conditions. Environ. Pollut..

[67] Shen H., Sun Y., Duan H., Ye J., Zhou A., Meng H., Zhu F., He H., Gu C. (2023). Effect of PVC microplastics on soil microbial community and nitrogen availability under laboratory-controlled and field-relevant temperatures. Appl. Soil Ecol..

[68] Zeng Q., Xiang J., Yang C., Wu J., Li Y., Sun Y., Liu Q., Shi S., Gong Z. (2023). Microplastics affect nitrogen cycling and antibiotic resistance genes transfer of sediment. Chem. Eng. J..

[69] Liu Y., Zhou H., Yan M., Liu Y., Ni X., Song J., Yi X. (2022). Toxicity of tire wear particles and the leachates to microorganisms in marine sediments. Environ. Pollut..

[70] Zhang X., Xia X., Dai M., Cen J., Zhou L., Xie J. (2021). Microplastic pollution and its relationship with the bacterial community in coastal sediments near Guangdong Province, South China. Sci. Total Environ..

[71] Huang Y., Li W., Gao J., Wang F., Yang W., Han L., Lin D., Min B., Zhi Y., Grieger K. (2021). Effect of microplastics on ecosystem functioning: Microbial nitrogen removal mediated by benthic invertebrates. Sci. Total Environ..

[72] Yu H., Liu M., Gang D., Peng J., Hu C., Qu J. (2022). Polyethylene microplastics interfere with the nutrient cycle in water-plant-sediment systems. Water Res..

[73] Lu X., Jiang X., Liu X. (2022). Response process and adaptation mechanism of estuarine benthic microbiota to polyvinyl chloride microplastics with and without phthalates. Sci. Total Environ..

[74] Zhu M., Yin H., Yuan Y., Liu H., Qi X., Ren Y., Dang Z. (2022). Discrepancy strategies of sediment abundant and rare microbial communities in response to floating microplastic disturbances: Study using a microcosmic experiment. Sci. Total Environ..

[75] Zhang W., Liu X., Liu L., Lu H., Wang L., Tang J. (2022). Effects of microplastics on greenhouse gas emissions and microbial communities in sediment of freshwater systems. J. Hazard. Mater..

[76] Chen C., Pan J., Xiao S., Wang J., Gong X., Yin G., Hou L., Liu M., Zheng Y. (2022). Microplastics alter nitrous oxide production and pathways through affecting microbiome in estuarine sediments. Water Res..

[77] Qin R., Su C., Liu W., Tang L., Li X., Deng X., Wang A., Chen Z. (2020). Effects of exposure to polyether sulfone microplastic on the nitrifying process and microbial community structure in aerobic granular sludge. Bioresour. Technol..

[78] Chen H., Wang Y., Sun X., Peng Y., Xiao L. (2020). Mixing effect of polylactic acid microplastic and straw residue on soil property and ecological function. Chemosphere.

[79] Dai H., Gao J., Wang Z., Zhao Y., Zhang D. (2020). Behavior of nitrogen, phosphorus and antibiotic resistance genes under polyvinyl chloride microplastics pressures in an aerobic granular sludge system. J. Clean. Prod..

[80] Azizi S.M.M., Hai F.I., Lu W., Al-Mamun A., Dhar B.R. (2021). A review of mechanisms underlying the impacts of (nano) microplastics on anaerobic digestion. Bioresour. Technol..

[81] Shen M., Zhang Y., Zhu Y., Song B., Zeng G., Hu D., Wen X., Ren X. (2019). Recent advances in toxicological research of nanoplastics in the environment: A review. Environ. Pollut..

[82] Huang J., Wen B., Miao L., Liu X., Li Z., Ma T., Xu L., Gao J., Chen Z. (2022). Microplastics drive nitrification by enriching functional microorganisms in aquaculture pond waters. Chemosphere.

[83] Ding J., Meng F., Chen H., Chen Q., Hu A., Yu C., Chen L., Lv M. (2022). Leachable additives of tire particles explain the shift in microbial community composition and function in coastal sediments. Environ. Sci. Technol..

[84] Nie Z., Wang L., Lin Y., Xiao N., Zhao J., Wan X., Hu J. (2022). Effects of polylactic acid (PLA) and polybutylene adipate-co-terephthalate (PBAT) biodegradable microplastics on the abundance and diversity of denitrifying and anammox bacteria in freshwater sediment. Environ. Pollut..

[85] Hao Z., He S., Wang Q., Luo Y., Tu C., Wu W., Jiang H. (2024). Nanoplastics enhance the denitrification process and microbial interaction network in wetland soils. Water Res..

[86] Sun Y., Duan C., Cao N., Ding C., Huang Y., Wang J. (2022). Biodegradable and conventional microplastics exhibit distinct microbiome, functionality, and metabolome changes in soil. J. Hazard. Mater..

[87] Riveros G., Urrutia H., Araya J., Zagal E., Schoebitz M. (2022). Microplastic pollution on the soil and its consequences on the nitrogen cycle: A review. Environ. Sci. Pollut. Res..

[88] Rogers K.L., Carreres-Calabuig J.A., Gorokhova E., Posth N.R. (2020). Micro-by-micro interactions: How microorganisms influence the fate of marine microplastics. Limnol. Oceanogr. Lett..

[89] Hirai H., Takada H., Ogata Y., Yamashita R., Mizukawa K., Saha M., Kwan C., Moore C., Gray H., Laursen D. (2011). Organic micropollutants in marine plastics debris from the open ocean and remote and urban beaches. Mar. Pollut. Bull..

[90] Shimao M. (2001). Biodegradation of plastics. Curr. Opin. Biotechnol..

[91] Galgani L., Tsapakis M., Pitta P., Tsiola A., Tzempelikou E., Kalantzi I., Esposito C., Loiselle A., Tsotskou A., Zivanovic S. (2019). Microplastics increase the marine production of particulate forms of organic matter. Environ. Res. Lett..

[92] Liu J., Xu G., Zhao S., He J. (2025). Microbiomes of coastal sediments and plastispheres shaped by microplastics and decabrominated diphenyl ether. Water Res..

[93] Chen M., Liu S., Bi M., Yang X., Deng R., Chen Y. (2022). Aging behavior of microplastics affected DOM in riparian sediments: From the characteristics to bioavailability. J. Hazard. Mater..

[94] Shen M., Ye S., Zeng G., Zhang Y., Xing L., Tang W., Wen X., Liu S. (2020). Can microplastics pose a threat to ocean carbon sequestration?. Mar. Pollut. Bull..

[95] Wang Q., Wangjin X., Zhang Y., Wang N., Wang Y., Meng G., Chen Y. (2020). The toxicity of virgin and UV-aged PVC microplastics on the growth of freshwater algae *Chlamydomonas reinhardtii*. Sci. Total. Environ..

[96] You X., You M., Lyu Y., Peng G., Sun W. (2022). Single and combined exposure to micro (nano) plastics and azithromycin disturbing the photosynthetic carbon fixation of *Synechocystis* sp.. Environ. Sci. Nano.

[97] Pinnell L.J., Turner J.W. (2019). Shotgun metagenomics reveals the benthic microbial community response to plastic and bioplastic in a coastal marine environment. Front. Microbiol..

[98] Hayden H.L., Drake J., Imhof M., Oxley A.P., Norng S., Mele P.M. (2010). The abundance of nitrogen cycle genes *amoA* and *nifH* depends on land-uses and soil types in South-Eastern Australia. Soil Biol. Biochem..

[99] Luo G., Jin T., Zhang H., Peng J., Zuo N., Huang Y., Han Y., Tian C., Yang Y., Peng K. (2022). Deciphering the diversity and functions of plastisphere bacterial communities in plastic-mulching croplands of subtropical China. J. Hazard. Mater..

[100] Luo G., Xue C., Jiang Q., Xiao Y., Zhang F., Guo S., Shen Q., Ling N. (2020). Soil carbon, nitrogen, and phosphorus cycling microbial populations and their resistance to global change depend on soil C:N:P stoichiometry. Msystems.

[101] Kim T., Lee J., Kim J., Oh J. (2015). Behavioral characteristics of phosphorus in sediments according to the forms of phosphorus. J. Ecol. Environ..

[102] Song X., Ding J., Zhang Y., Zhu M., Peng Y., Wang Z., Pan G., Zou H. (2024). New insights into changes in phosphorus profile at sediment-water interface by microplastics: Role of benthic bioturbation. J. Hazard. Mater..

[103] Zhou Z., Tran P.Q., Cowley E.S., Trembath-Reichert E., Anantharaman K. (2025). Diversity and ecology of microbial sulfur metabolism. Nat. Rev. Microbiol..

[104] Dombrowski N., Teske A.P., Baker B.J. (2018). Expansive microbial metabolic versatility and biodiversity in dynamic Guaymas Basin hydrothermal sediments. Nat. Commun..

